# Regulation of Boundary Cap Neural Crest Stem Cell Differentiation After Transplantation

**DOI:** 10.1002/stem.77

**Published:** 2009-07

**Authors:** Hakan Aldskogius, Christian Berens, Nadezda Kanaykina, Anna Liakhovitskaia, Alexander Medvinsky, Martin Sandelin, Silke Schreiner, Michael Wegner, Jens Hjerling-Leffler, Elena N Kozlova

**Affiliations:** a Department of Neuroscience, Neuroanatomy, Uppsala University Biomedical CenterUppsala, Sweden; b Department of Biology, University of Erlangen-NurembergErlangen, Germany; c Institute for Stem Cell Research, University of EdinburghEdinburgh, United Kingdom; d Department of Biochemistry, University of Erlangen-NurembergErlangen, Germany; e Division of Molecular Neurobiology, Department of Medical Biochemistry and Biophysics, Karolinska InstitutetStockholm, Sweden

**Keywords:** Sensory neuron, In vitro, Mouse, Transcription factor, Transgene, Tet-system

## Abstract

Success of cell replacement therapies for neurological disorders will depend largely on the optimization of strategies to enhance viability and control the developmental fate of stem cells after transplantation. Once transplanted, stem/progenitor cells display a tendency to maintain an undifferentiated phenotype or differentiate into inappropriate cell types. Gain and loss of function experiments have revealed key transcription factors which drive differentiation of immature stem/progenitor cells toward more mature stages and eventually to full differentiation. An attractive course of action to promote survival and direct the differentiation of transplanted stem cells to a specific cell type would therefore be to force expression of regulatory differentiation molecules in already transplanted stem cells, using inducible gene expression systems which can be controlled from the outside. Here, we explore this hypothesis by employing a tetracycline gene regulating system (Tet-On) to drive the differentiation of boundary cap neural crest stem cells (bNCSCs) toward a sensory neuron fate after transplantation. We induced the expression of the key transcription factor Runx1 in Sox10-expressing bNCSCs. Forced expression of Runx1 strongly increased transplant survival in the enriched neurotrophic environment of the dorsal root ganglion cavity, and was sufficient to guide differentiation of bNCSCs toward a nonpeptidergic nociceptive sensory neuron phenotype both in vitro and in vivo after transplantation. These findings suggest that exogenous activation of transcription factors expression *after* transplantation in stem/progenitor cell grafts can be a constructive approach to control their survival as well as their differentiation to the desired type of cell and that the Tet-system is a useful tool to achieve this.

## INTRODUCTION

The use of stem/progenitor cells is an attractive avenue for cell replacement strategies in the treatment of neurological disorders and traumatic injuries. However, to generate neurons, and particularly of a specific desired type, from the transplanted stem cells still poses a significant challenge. The current state of the art in neural stem cell transplantation is to differentiate stem cells in vitro into the required cell type and to subsequently transplant an enriched population of these cells into the injured or diseased nervous system. However, this might not be the optimal protocol for every application, since the time window for a successful transplantation may be narrow for some types of stem/progenitor cells and their fate after transplantation remains unpredictable. As a result, the transplanted neural stem cells survive transplantation poorly or undergo inappropriate or incomplete differentiation [1–3]. An alternative approach to promote differentiation of stem cells would focus on initiating this process *after* transplantation. The aim of this study was to investigate if the exogenous induction of key transcription factor(s) *after* transplantation could be effective in inducing subtype-specific differentiation of transplanted stem cells. Here, we explore this approach in a model system of boundary cap neural crest stem cell (bNCSC) transplantation to the dorsal root ganglion (DRG) cavity.

We previously developed a method of transplantation to the DRG cavity that allows the identification of transplanted cells by their specific location, and demonstrated that the transplanted sensory neurons established functional connections with the spinal cord [4–6]. When transplanted in this system, mouse forebrain neural stem/progenitor cells (NSPCs) predominantly differentiated into glial cells [7], while human NSPCs differentiated into neurons, although they remained immature for up to 3 months post-transplantation [8]. In the present study, we prepared bNCSCs for transplantation from the embryonic 11,5 DRGs, including the boundary cap (b), a transient neural crest derived structure giving rise to the last wave of DRG neurons in development [9–11]. The bNCSCs are multipotent stem cells that have been shown to generate sensory neurons in vitro [12, 13] and glial cells in vivo after transplantation [14]. Here, we explore whether conditionally induced expression of the key transcription factor Runx1 in Sox10-expressing cells from transgenic mice can guide the differentiation of such peripherally transplanted bNCSCs toward a nociceptor neuron phenotype.

Differentiation of neural stem cells is controlled by the combined action of external signals and sequentially expressed transcription factors, with some of these representing master regulators [15, 16]. We chose to control Runx1 using the Sox10 pattern of expression, since Runx1 had been suggested to play a key role in the initial stage of differentiation of NCSCs into neurons [11].

Sox10 or SRY-box containing gene 10, is a high mobility group transcription factor expressed in all neural crest (NC) cells and is involved in several aspects of NC development [17, 18]. Importantly, it maintains NC cell multipotency [19] and hence, its downregulation is a prerequisite for neuronal differentiation [20, 21]. We employed the tetracycline gene regulating system (Tet-On) [22] for exogenous induction of Runx1 overexpression in bNCSCs in vitro and after transplantation. This approach allowed us to use Sox10-rtTA mice [23] to specifically target Sox10-expressing cells, since only they will respond with TRE-Runx1 activation. We find that exogenous activation of Runx1 expression in Sox10 expressing bNCSCs promotes survival and induces specific differentiation toward nonpeptidergic nociceptive-type sensory neurons in vitro and after transplantation.

## MATERIALS AND METHODS

### Animals and Genotyping

*Sox10-rtTA2^S^-M2* mice, which contain a second-generation reverse tetracycline-controlled transactivator (rtTA2^S^-M2) knocked into the genomic *Sox10* locus, have been previously described along with protocols for their genotyping [23]. Transgenic heterozygous C57BL/6-β-actin (CAG)-enhanced green fluorescent protein (EGFP) mice (Jackson Laboratories, Bar Harbor, ME, http://www.jax.org) were used as previously described [7]. As control to exclude any doxycycline (DOX)-mediated effects on bNCSC differentiation in the transplants, we used neurospheres from ROSA26-rtTA/HPRT-IRES-EGFP mice (generated in the Medvinsky laboratory) in which expression of EGFP was activated by Tet-On. ROSA26-rtTA/HPRT-IRES-EGFP mice were generated from Ainv15 embryonic stem (ES) cells [24] These cells contain the rtTA transgene targeted into the ROSA26 locus and a homing site for a single LoxP targeting site upstream of the HPRT locus. cDNA targeted into the homing site is driven by a Tet-dependent promoter. The ES cell line was generated by targeting IRES-EGFP. ES clones that showed high levels of EGFP expression on addition of DOX were selected for blastocyst injections.

For experiments aimed at evaluating postgrafting bNCSC survival and extension of the transplanted cells, embryos were obtained by breeding heterozygous *EGFP* male and heterozygous *Sox10-rtTA2^S^-M2* (Sox10^+/rtTA^) female mice. Embryos with EGFP expression were identified using fluorescence microscopy and genotyped for the presence of *Sox10-rtTA2^S^-M2*. All procedures were approved by the Regional Ethical Committee for Research on Animals and carried out according to the guidelines of the Society for Neuroscience.

### Culture of bNCSCs

bNCSCs were isolated in a semiclonal fashion from Sox10^+/rtTA^ or from CAG-EGFP:Sox10^+/rtTA^ embryos on embryonic day (E) 11, as described previously [12]. Briefly, the DRGs along with boundary caps were mechanically separated from the isolated spinal cord and mechanoenzymatically dissociated using Collagenase/Dispase (1 mg/ml) and DNase (0.5 mg/ml) for 30 minutes at room temperature. Cells were plated at 0.5–1 × 10^5^ cells/cm^2^ in N2 medium containing B27 (Gibco, Grand Island, NY, http://www.invitrogen.com) as well as EGF and basic fibroblast growth factor (R&D Systems, Minneapolis, http://www.rndsystems.com; 20 ng/ml, respectively). After 12 hours, nonadherent cells were removed together with half of the medium before adding fresh medium. The medium was changed every other day (50% of the medium replaced with fresh medium) until neurospheres could be observed after approximately 2 weeks of culture. Nonpassaged neurospheres between 2 and 3 weeks in culture were used for subsequent experiments.

### Quantitative Reverse-Transcription Polymerase Chain Reaction

Primers used were Sox10F: TCTACACTGCCATCTCTGAC (nt: 1,691–1,710) and Sox10R: CTCCTCCACTGCCAAGC (nt: 1,871–1,887), product length 197 bp ((Acc. no. gi|149266994). EYFPF: GACGTAAACGGCCACAAGTT and EYFPR GTCCTCCTTGAAGTCGATGC, product length 339 bp; Runx1F: CTCTGCTCCGTGCTGCC and Runx1R: GTCATTAAATCTCGCAACC, product length 189 bp. Total bNCSC RNA was extracted and transcribed with a kit (Bio-Rad, Hercules, CA, http://www.bio-rad.com) into cDNA using random priming with MMLV reverse transcriptase. cDNA from 5 ng of total RNA was used for each reaction and reverse-transcription polymerase chain reaction (RT-PCR) performed as described previously [25]. All data were analyzed in Microscoft Excel.

### Nucleofection of DNA and Activation of eYFP/Runx1 Expression in Transfected Cells

**Figure 1:**
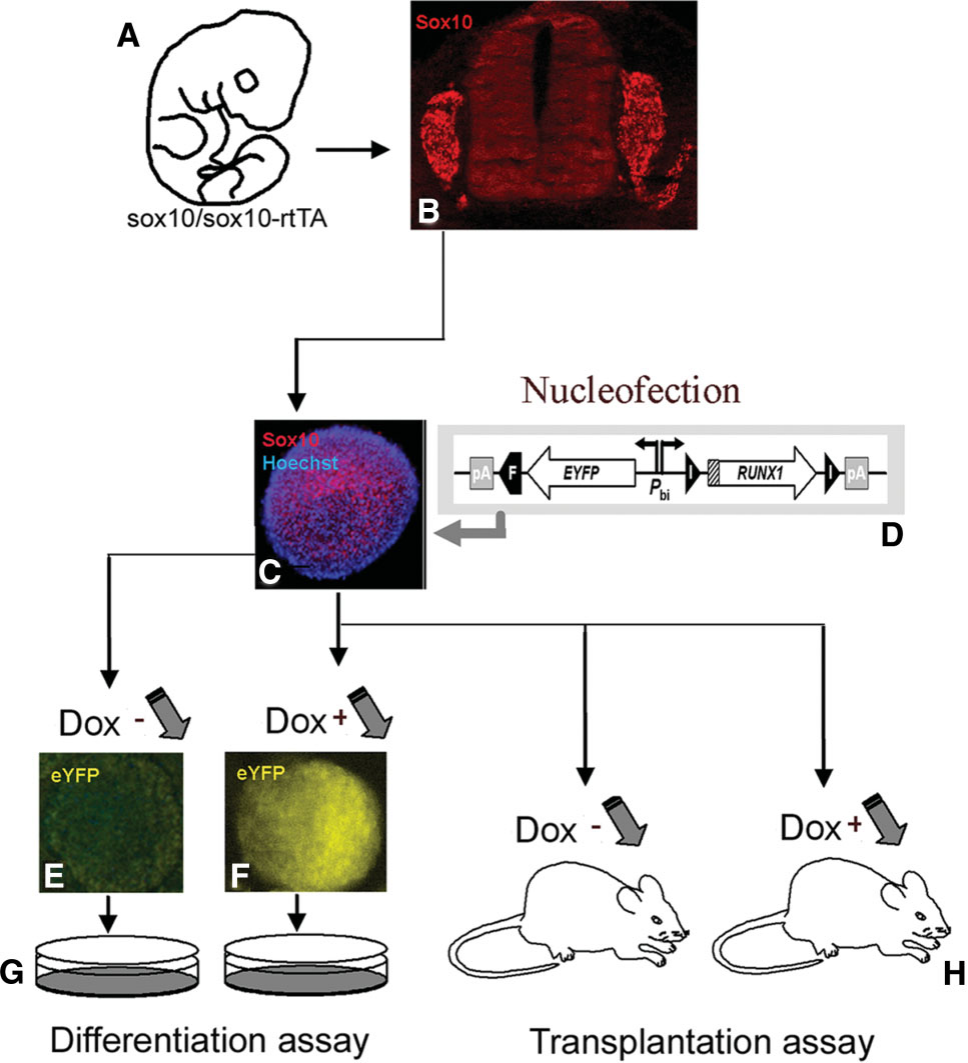
Overview of experiments. Sox10^+/rtTA^ embryos at E11.5 **(A):** express Sox10 (red) in the dorsal root ganglia **(B)** as well as in newly formed boundary cap neural crest stem cell (bNCSC) neurospheres **(C)**. The isolated bNCSCs are transfected with the TRE_bi_-Runx1-eYFP vector **(D)** containing a bidirectional promoter, which on DOX treatment induces expression of both Runx1 and EYFP. DOX-activated expression of Runx1/EYFP in bNCSC neurospheres **(E)** two days after transfection and 1 day after DOX activation before differentiation assay **(F)** and transplantation **(G)**. Nuclear labeling with Hoechst is shown in blue. Abbreviation: DOX, doxycycline.

We used the construct described in Figure 1D. Nucleofection of 3-weeks-old *Sox10^+/rtTA^* neurospheres was performed with an Amaxa Biosystems Nucleofector according to the manufacturer's instructions for the Optimal Nucleofector Program A33. The level of transfection and the efficacy of the Tet-On system were assessed by analyzing the EYFP expression in DOX (5 μg/ml)-treated and DOX-untreated cultures. DOX was added to cultured neurospheres 24 hours after transfection and replaced every third day when the culture medium was changed. The EYFP fluorescence was monitored in an inverted fluorescence microscope. To verify the validity of EYFP expression as a measure of Runx1 upregulation, the expression of Runx1 and EYFP in DOX-treated and nontreated cultures was assessed with RT-PCR.

### In Vitro Differentiation Assay

DOX-treated EYFP-expressing neurospheres were picked up by pipette under the fluorescence microscope, and plated at a density of 1.2 × 10^3^ cells on a poly-D-lysine (50 μg/ml)/laminin (20 ng/ml)-coated coverslip and maintained in Dulbecco's modified Eagle's medium-F12/neurobasal medium supplemented with N2, B27, 0.1 mM nonessential amino acids and 2 mM sodium pyruvate. Nontreated neurospheres were used in parallel as control. The medium was replenished and DOX was added every third day. After 1 or 2 weeks, cells were fixed with 4% phosphate buffered paraformaldehyde. Each experiment was repeated at least six times.

### Transplantation and Post-Grafting Treatment

Adult C3H mice (Mollegard, Denmark) were anesthetized by intraperitoneal injection of xylazine (Rompun®vet.; http://www.bayer.com) and ketamine (Ketaminol®vet.; http://www.intervet.com) (10 and 100 ng per gram body weight, respectively). The transplantation procedure was described previously [4–7]. Briefly, the left L4 DRG was exposed via a partial laminectomy and carefully removed, leaving the ventral root intact. Four to five Sox10^+/rtTA^ bNCSC neurospheres transfected with TRE_bi_-EYFP-Runx1 or the same amount of neurospheres produced from ROSA26-rtTA/HPRT-IRES-EGFP mice were placed into the empty DRG cavity to an approximate total number of 10,000 cells/transplant, the dorsal root and the spinal nerve were attached to the collection of bNCSCs and the wound closed in layers.

All recipients (*n* = 41) received daily intraperitoneal injections of cyclosporin (Sandimmun, Novartis International, Basel, Switzerland, http://www.novartis.com; 15 mg/kg body weight). The experimental groups (*n* = 12) with Sox10^+/rtTA^:TRE_bi_-EYFP-Runx1 donor cells received DOX in their drinking water (3 g/l with 50 g/l sucrose) from the first day after surgery and through the entire post-transplantation period while the control group with TRE_bi_-EYFP-Runx1 donor cells (*n* = 6) received pure drinking water.

In an additional experiment to evaluate transplant size and overall survival of transplanted bNCSCs, CAG-EGFP:Sox10^+/rtTA^ bNCSCs ubiquitously expressing EGFP were used (*n* = 6 treated; *n* = 3 control) with the same treatment.

As a control to exclude effects from the DOX treatment itself, we included nontransfected (wt) EGFP bNCSCs treated, or not treated with DOX (*n* = 3 for each group), nontransfected sox10-rtTA treated with DOX (*n* = 3) and ROSA26-rTA/HPRT-IRES-EGFP treated with DOX (*n* = 5).

For bromodeoxyuridine (BrdU) labeling, six mice with neurospheres harboring Sox10^+/rtTA^:TRE_bi_-EYFP-Runx1 transgenes were injected with BrdU intraperitoneally once every day (10 mg/ml; 200 μl) from day 1 after transplantation and for a time-period of 2 weeks. Three of these mice were given DOX in their drinking water from the first post-transplantion day and the other three mice were given DOX with a delay of 1 week postgrafting.

### Processing of Transplant Tissue

One month after transplantation, the animals were reanesthetized and perfused with phosphate-buffered picric acid–formalin. The graft sites, together with the attached dorsal root and spinal nerve, as well as the corresponding part of the spinal cord were removed, placed in cold fixative for 4 hours, and cryoprotected overnight in phosphate-buffered saline (PBS) containing 15% sucrose. Tissue was sectioned using a cryostat at 9 μm thickness and stored at −20°C for later immunohistochemical processing.

### Immunohistochemistry

Immunolabeling was performed as described previously [26]. Primary antibodies were anti-βIII-tubulin (bTUB; mouse monoclonal; Covance, Princeton, NJ, http://www.covance.com, 1:500; rabbit polyclonal; Covance, 1:1,000), anti-calcitonin gene-related peptide (CGRP; rabbit polyclonal; Chemicon, Temecula, CA, http://www.chemicon.com, 1:4,000), anti-peripherin (mouse monoclonal; Sigma-Aldrich, http://www.sigmaaldrich.com; 1:1000), anti-glial fibrillary acidic protein (GFAP; rabbit polyclonal; DAKO, Glostrup, Denmark, http://www.dako.com; 1:1,000), anti-RET (goat polyclonal; R&D Systems, Minneapolis, MN, http://www.rndsystems.com; 1:20), anti-Sox10 (guinea pig polyclonal; 1:1,000), anti-TrkA (rabbit polyclonal; Santa Cruz, CA, http://www.scbt.com; 1:200), RT97 (mouse monoclonal; Immunkemi, Jarfalla, Sweden, http://www.immunkemi.se; 1:500), anti-P2X3 (rabbit polyclonal; Millipore, Billerica, MA, http://www.millipore.com; 1:400), and anti-EGFP (mouse monoclonal; Invitrogen, Carlsbad, CA, http://www.invitrogen.com, 1:400). Secondary antibodies (Jackson Immunoresearch Laboratories, West Grove, PA, http://www.jacksonimmuno.com) were diluted in PBS with 0.3% Triton X-100 and 0.1% sodium azide: AMCA-conjugated donkey anti-rabbit and anti-mouse, Cy3-conjugated donkey anti-mouse, FITC-conjugated donkey anti-rabbit, TRITC-conjugated rabbit anti-goat. For isolectin B4 (IB4) labeling, cultures/slides were incubated in FITC-conjugated *Griffonia Simplicifolia* Agglutinin IB4 (Vector Laboratories, Burlingame, CA, http://www.vectorlabs.com; 1:100) for 4 hours. Sections were rinsed 3 × 15 minutes in PBS, (the second wash included Hoechst 33342; 11 ng/ml; Molecular Probes, Eugene, OR, http://probes.invitrogen.com) and then mounted in a mixture of PBS and glycerol (1:1; v/v) containing 0.1 M propyl gallate. Incubations without primary antibodies were performed to exclude unspecific labeling from secondary antibodies.

### In Situ Hybridization

In situ hybridization for RET mRNA was performed on DOX-treated and DOX-untreated Sox10^+/rtTA^:TRE_bi_-EYFP-Runx1 and Sox10^+/rtTA^:TRE_bi_-EYFP-Runx1/EGFP transplants with a RET probe (kindly provided by Dr. Q. Ma) and according to refs. [27, 28]. The hybridization signal was detected using an alkaline phosphatase-conjugated antibody (1:2,000; Roche, Indianapolis, IN) and 5-bromo-4-chloro-indolyl-phosphate/nitroblue-tetrazolium staining.

### Microscopic Analysis

Immunolabeled cultures and sections were analyzed in a Nikon Eclipse E800 fluorescence microscope. For photography, a Nikon DXM1200F digital camera system was used. The proportion of neuronal cells expressing subtype-specific sensory markers was estimated in the following combinations: bTUB/IB4; peripherin/CGRP, bTUB/RET. Cells were counted under the 20× objective with the aid of a square ocular frame (side 0.11 mm). Neurite outgrowth in the in vitro differentiation assay was analyzed using a computer-based procedure [29]. Briefly, 10 photographs (20× objective) were taken of bTUB-labeled cultures (*n* = 6 treated; *n* = 6 control) and placed in a grid frame on the computer screen. The number of intersections between neurites and grid lines in relation to the number of cells within the frame was analyzed and used as a measure of neurite outgrowth/cell.

Every sixth slide from transplants with Sox10^+/rtTA^:TRE_bi_-EYFP-Runx1 and ROSA26-rtTA/HPRT-IRES-EGFP neurospheres was immunostained and cells containing nuclei were analyzed under the 20× objective with the aid of a square ocular frame (side 0.11 mm). For the combination IB4/CGRP/RT97 the number of cell bodies labeled with either one of these markers was counted. For evaluation of transplant size and bNCSC survival, every fifth section from EGFP transplants was photographed. The NIH software ImageJ (Rasband, 1997, available at http://rsb.info.nih.gov/ij) was used to measure transplant areas. The transplant volume estimate was calculated according to the formula *A* = *TK*[∑(S_1_ to S_n_)], where *T* is the thickness of the section (*T* = 9 μm), *K* is the number of sections between the measured areas (*K* = 5) and *S* is the area of the transplant on the sections from 1 to *N*. To generate three-dimensional images of transplants, serial sections from transplants with eGFP expressing donor cells were photographed and digitalized (see earlier), manually aligned in Photoshop, and processed with the VolView 2.0 software (KitWare, Clifton Park, NY).

For an estimate of the cell numbers in each transplant, EGFP+ cells with nuclei were counted in every fourth section. To correct for possible differences in nuclear size in different transplants, the average nuclear diameter was analyzed by measuring 30 randomly selected nuclei. The number of neurons counted was multiplied by section separation to give a total estimated number of profiles (*n*). This number was multiplied by section thickness (*T*), divided by *T* plus the average diameter of the nuclei (*D*) to give the neuronal number (*N*); *n* = *n* × *T*/(*T* + *D*) [30, 31].

### Statistics

Data were analyzed using a two-tailed unpaired Student's *t* test.

## RESULTS

### Sox10 Expression in bNCSCs In Vitro

An overview of the experimental setup is shown in Figure 1. The bNCSC neurospheres were produced from *Sox10^+/rtTA^* mouse embryos. Consistent with previous results [20], immunohistochemical analysis showed that Sox10 was expressed in E11 DRGs of Sox10^+/rtTA^ mice (Fig. 1B). Sox10 expression was also found in newly formed bNCSC neurospheres (Fig. 1C) and the expression with regards to passage and days in culture was assayed by RT-PCR (supporting information Fig. 1). As seen by RT-PCR Sox10 was initially highly expressed, but this expression declined after 4 to 5 weeks in culture. Thus for the experiments of this study we did not use bNCSCs older than 3 weeks. To evaluate the expression of transgenic Runx1 in DOX-activated neurospheres, we performed RT-PCR and confirmed that the expression of Runx1 in DOX-activated neurospheres paralleled the time course of EYFP fluorescence (supporting information Fig. 2).

### Differentiation of Sox10^+/rtTA^:TRE_Bi_-Runx1-EYFP bNCSCs In Vitro

We next assessed how forced Runx1 expression influences the differentiation of bNCSCs in vitro. We first analyzed the level of EYFP expression in transfected bNCSC neurospheres to determine the level of transfection and DOX-induced transcription. Sox10^+/rtTA^ neurospheres were transfected with the TRE_bi_-Runx1-EYFP expression vector (Fig. 1D). The cells were divided in two groups and DOX was added to the medium of one of these groups 24 hours after transfection. The expression of Runx1 in DOX-treated and control neurospheres was monitored by the concomitant induction of EYFP expression from the bidirectional TRE promoter (Fig. 1E, 1F). EYFP fluorescence was observed in around 60% of the DOX-treated neurospheres, after 24–48 hours of DOX administration (supporting information Fig. 2). The percentage of EYFP-expressing cells in individual neurospheres varied between approximately 10% and 90%. In DOX-untreated neurospheres, the expression of EYFP was extremely low and detected only in about 0.3%–1% of the total number of cells. EYFP/Runx1 expression in DOX-treated neurospheres gradually declined in intensity and disappeared after 2–3 weeks in culture which is in agreement with the cessation of Sox10 expression (data not shown).

Neurospheres were plated on D-polylysine-laminin coated coverslips 2 days after transfection in a medium without mitogens to allow for differentiation. No neurotrophic factors were added to decrease the risk of a Runx1-mediated effect being masked by differentiation directed by extrinsic factors. DOX was added in the treated group throughout the experiment and EYFP expression was monitored. The EYFP expression rapidly declined in differentiating cells (supporting information Fig. 3). However, in a few cells it was retained for up to 2 weeks, possibly due to rare and random stable integration events leading to poor inactivation of the Tet-promoter once activated. This time frame of 2 weeks allowed us to visualize the gradual differentiation of EYFP-expressing bNCSCs to cells with a typical DRG neuron morphology (supporting information Fig. 4).

**Figure 2:**
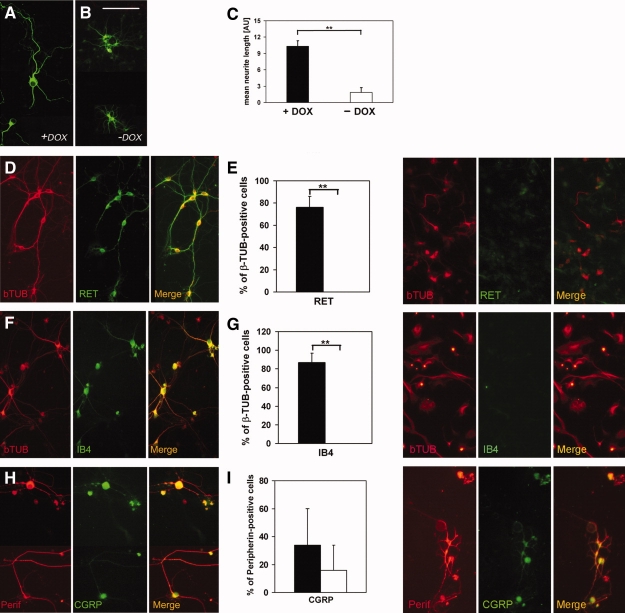
DOX-activated Runx1 expression induced differentiation of boundary cap neural crest stem cells to dorsal root ganglion neurons in vitro. DOX-induced expression of Runx1 leads to significantly increased neurite outgrowth. DOX-treated **(A)** and control **(B)** cultures, quantified in (**C**; *p* = 3.6 × 10^−8^). Panels **(D)**, **(F)**, and **(H)** show DOX-treated cultures to the left and DOX-untreated cultures to the right of the graphs. The quantitative analyses of DOX-treated and DOX-untreated cultures are shown in graphs **(E)**, **(G)**, and **(I)**. RET expression is induced in βIII-tubulin (bTUB)+ cells in DOX-treated, but not in DOX-untreated cultures (panel **D**; quantified in **E**) (*p* = 1.8 × 10^−4^). Also isolectin B4 binding by bTUB+ cells was induced in DOX-treated, but not in DOX-untreated cultures (panel **F**; quantified in **G**) (*p* = 3.6 × 10^−6^). Conversely, there was no significant difference in calcitonin gene-related peptide-labeled cells in DOX-treated compared with DOX-untreated cultures (panel **H**; quantified in **I**) (*p* = .21). Scale bar = 100 μm. Abbreviation: DOX, doxycycline.

Cultures were fixed at the end of the experiment (2 weeks) and labeled with markers for sensory neuron subtypes, with particular focus on DRG neurons previously reported to be Runx1-dependent. During early development, Runx1 is expressed in the largest subpopulation of DRG neurons, the small diameter nociceptive class [26, 32]. In addition, Runx1 has been reported to induce neurite outgrowth from DRG neurons [32]. In agreement with these findings, we found that DOX-induced Runx1 expression increased neurite outgrowth in bTUB+ cells in culture (Fig. 2A, 2B; quantified in c; *p* = 3.6 × 10^−8^). Looking at the subtype markers, we found abundant induction of the markers IB4 and glial cell line-derived neurotrophic factor (GDNF) family coreceptor RET in the DOX-treated cultures, while they were completely absent in the control group (Fig. 2D–2G; in (e) *p* = 3.6 × 10^−6^ and (g) *p* = 1.8 × 10^−4^). These markers are characteristic for nonpeptidergic nociceptor DRG neurons. In contrast, CGRP, a marker for small peptidergic nociceptive neurons, showed no obvious difference between DOX-treated and control cultures (Fig. 2H, 2I; *p* = .21). These data suggest that ectopic expression of Runx1 is sufficient to drive differentiation of bNCSCs toward nonpeptidergic DRG neurons in the absence of neurotrophic factors in vitro.

### Differentiation of Sox10^+/rtTA^:TRE_Bi_-Runx1-EYFP bNCSCs After Transplantation

After determining that forced overexpression of Runx1 in Sox10-expressing bNCSCs in vitro can induce differentiation toward the nonpeptidergic nociceptor phenotype, we examined whether this could also influence their differentiation in vivo after transplantation. To this end, we used TRE_bi_-Runx1-EYFP transfected neurospheres, taken from cultures in which DOX-induced EYFP expression had been confirmed in samples (the grafted cells however, had not been treated with DOX prior to transplantation), in a transplantation experiment. The left L4 DRG was removed and the transfected bNCSCs were grafted in its place with nerve root endings being reattached to the graft. One group of graft recipients received DOX in their drinking water whereas the control group received tap water. One month later, the L4 cavity containing the grafted cells was harvested for further investigation.

**Figure 3:**
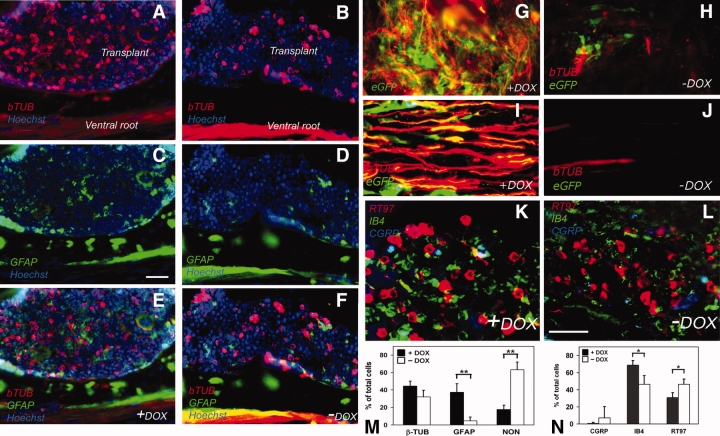
bNCSC grafts one month after transplantation to the L4 dorsal root ganglion cavity. **(A–F):** Immunostainings for βIII-tubulin (bTUB; red) and GFAP (green) together with nuclear marker Hoechst (blue). **(A, B):** bTUB staining shows slightly more positive cells in the DOX-treated animals. **(C, D):** Increased GFAP staining in the DOX-treated animals. Note the difference in morphology of positive cells and the overall structure between the two treatments. **(G–J):** Growth of bTUB+ fibers from EGFP expressing bNCSCs is extensive within the graft **(G)** and in the associated dorsal root **(I)** of DOX-treated **(G, I)** compared with DOX-untreated **(H, J)** transplants. bTUB+ neurites from DOX-treated bNCSC transplants intermingle with bTUB+ host fibers in the dorsal root **(I)**. **(K, L):** Immunostaining for CGRP (blue), RT97 (red), and isolectin B4 (IB4; green). A small number of CGRP+ cells occur in both conditions. There was a decreased number of RT97+ cells while IB4 binding was increased by DOX-activated Runx1 expression. **(M):** Quantification of bTUB (*p* = .085) and GFAP stainings (*p* = 4.7 × 10^−4^). **(N):** Quantification of CGRP (*p* = .32), IB4 (*p* = .0048), and RT97 (*p* = .0022) stainings. Scale bar = 50 μm. **(A–H; K, L)**; 25 μm **(I, J)**. Abbreviations: bTUB, βIII-tubulin; CGRP, calcitonin gene-related peptide; DOX, doxycycline; GFAP, glial fibrillary acidic protein.

We first estimated the relative extent of neuron versus glial differentiation in DOX-treated and control transplants, using immunohistochemistry for bTUB and GFAP in combination with nuclear counterstaining (Fig. 3A–F). The proportion of bTUB+ cells was higher in DOX-treated transplants (although not significantly, *p* = .08) whereas that of GFAP+ cells was significantly higher compared with DOX-untreated transplants (*p* = 4.7 × 10^−4^). In DOX-untreated transplants, in contrast, the proportion of bTUB/GFAP negative cells was greater (Fig. 3I, *p* = 1.6 × 10^−5^).

To assess the influence of DOX treatment on neurite outgrowth from the transplanted cells, we analyzed transplants, dorsal roots associated with the transplanted tissue, and corresponding areas (L4 and L5) in the spinal cord of DOX-treated and DOX-untreated EGFP/Sox10-rtTA2^S^-M2 (Sox10+/rtTA) mice. DOX-treated Runx1-induced EGFP+ cells displayed a more mature morphology than DOX-untreated cells (Fig. 3G, 3H) and showed extensive neurite outgrowth into the host dorsal roots (Fig. 3I, 3J). However, neurites extending from DOX-treated transplants did not fully reach the host spinal cord.

**Figure 4:**
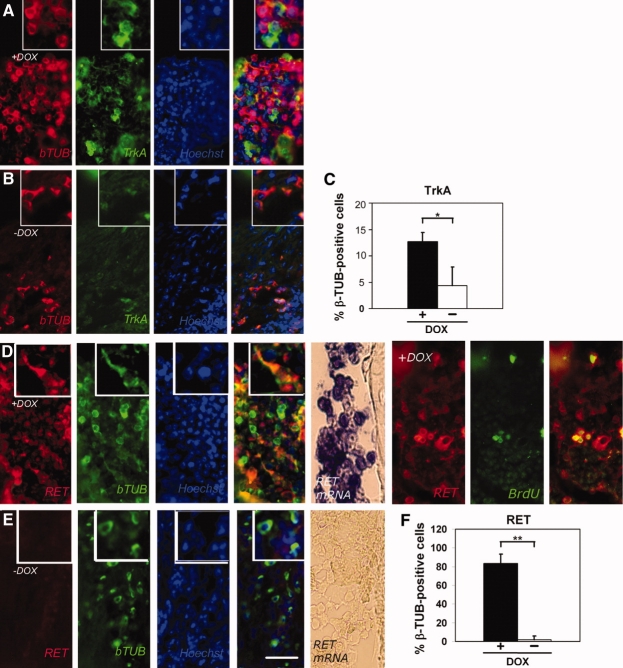
Immunostainings and quantification of TrkA and RET in transplants. Staining for bTUB (red) and TrkA (green) show an increased number of TrkA+ cells after DOX treatment (panel **A**) when compared with the control (panel **B**). Some TrkA is expressed in bTUB+ cells but most TrkA staining is associated with bTUB negative cells. **(C):** Quantification of the increase of TrkA positive cells among the bTUB+ population (*p* = .0034). Staining of RET (red) in DOX-treated (panel **D**) and DOX-untreated (panel **E**) transplants. In DOX-treated transplants, the majority of RET expression (panel **D**; red) is associated with bTUB+ (green) cells. In situ hybridization for RET mRNA shows extensive labeling in DOX-treated (panel **D**), and absence of labeling in DOX-untreated (panel **E**) transplants. BrdU-labeled cells, some of which express RET are shown in panel **D** (right). **(F):** Quantification of immunolabeled cells **(D, E)** showing the almost complete absence of RET positive cells in the control (*p* = 3.4 × 10^−7^). Nuclear labeling with Hoechst is shown in blue. Scale bar (**E**) = 50 μm. Abbreviations: bTUB, βIII-tubulin; BrdU, bromodeoxyuridine; DOX, doxycycline.

**Figure 5:**
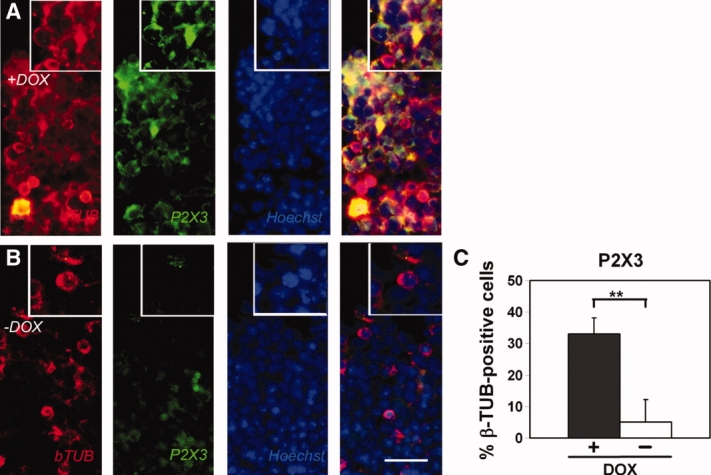
Immunostainings and quantification of the ATP receptor P2X3 in transplants. Staining for bTUB (red) and P2X3 (green) shows an increased number of P2X3+ cells after DOX treatment **(A)** when compared with the control **(B)**. **(C):** Quantification of the increase of positive cells among the bTUB+ population (*p* = .0034; *n* = 6). Scale bar (**B**) = 50 μm. Abbreviations: bTUB, βIII-tubulin; DOX, doxycycline.

We next characterized the neuronal phenotypes in transplants from DOX-treated and nontreated recipients, using sensory neuron subtype-specific markers. Transplants from DOX-treated mice were characterized by an increase in IB4-labeled (nonpeptidergic) cells (Fig. 3G, 3H, 3J; *p* = .005). The percentage of cells immunoreactive for RT97 (antibody labeling the phosphorylated form of neurofilament heavy-chain specific for mechanosensitive cells) was significantly decreased (Fig. 3J; *p* = .002). The percentage of CGRP+ cells also showed a decreasing trend, but was not shown to be significant (Fig. 3J; *p* = .31). We also investigated the expression of TrkA and RET in the bTUB+ cells in transplants from DOX-treated and control mice (Fig. 4). The amount of cells expressing both markers was significantly increased in DOX-treated transplants (Fig. 4 C and 4F; *p* = .003 and *p* = 3.4 × 10^−7^, respectively). Extensive expression of RET in DOX-treated transplants was confirmed by in situ hybridization for RET mRNA (Fig. 4D). However, while TrkA was mostly expressed in non-neuronal bTUB-negative cells (Fig. 4A, 4B) the majority of RET-expressing cells were bTUB+ (Fig. 4D, 4E). In addition, we assayed for the presence of the ATP receptor P2X3 which is expressed in a subset of the non-peptidergic nociceptive neurons [33] that expresses Mas-related G protein-coupled receptor D and peripherin and innervates the superficial layers of the skin [34]. P2X3 was increased in the neuronal (bTUB+) population by the induction of Runx1 expression (Fig. 5A–C; *p* = 1.3 × 10^−4^). The relative proportion of P2X3-expressing cells was smaller than the fraction of RET-expressing cells suggesting that, similar to the normal DRG, only a subset of the RET+ cells express P2X3 (Figs. 4F, 5C). These data suggest that using the DOX-inducible system to overexpress Runx1 in Sox10+ bNCSCs increases the differentiation into a non-peptidergic nociceptive phenotype, possibly at the expense of other sensory neuronal cell types. After BrdU pulse labeling in the early post-transplantation period, some RET-expressing neurons were also BrdU+ indicating that this population indeed originated from proliferating bNCSCs (Fig. 4D).

To asses the effect of DOX treatment on transplant survival and differentiation, we transplanted nontransfected CAG-EGFP/Sox10-rtTA, Sox10-rtTA, and ROSA26-rtTA/HPRT-IRES-EGFP bNCSC neurospheres. We found no difference in survival and differentiation between DOX-treated nontransfected transplants and ROSA26-rtTA/HPRT-IRES-EGFP treated with DOX compared with the DOX-untreated Runx1 transfected group (supporting information Fig. 8) Particularly striking was the complete absence of RET (supporting information Fig. 8) and P2X3 expressing cells in the control groups.

### Survival of CAG-EGFP:Sox10^+/rtTA^:TRE_Bi_-Runx1-EYFP bNCSCs After Transplantation

**Figure 6:**
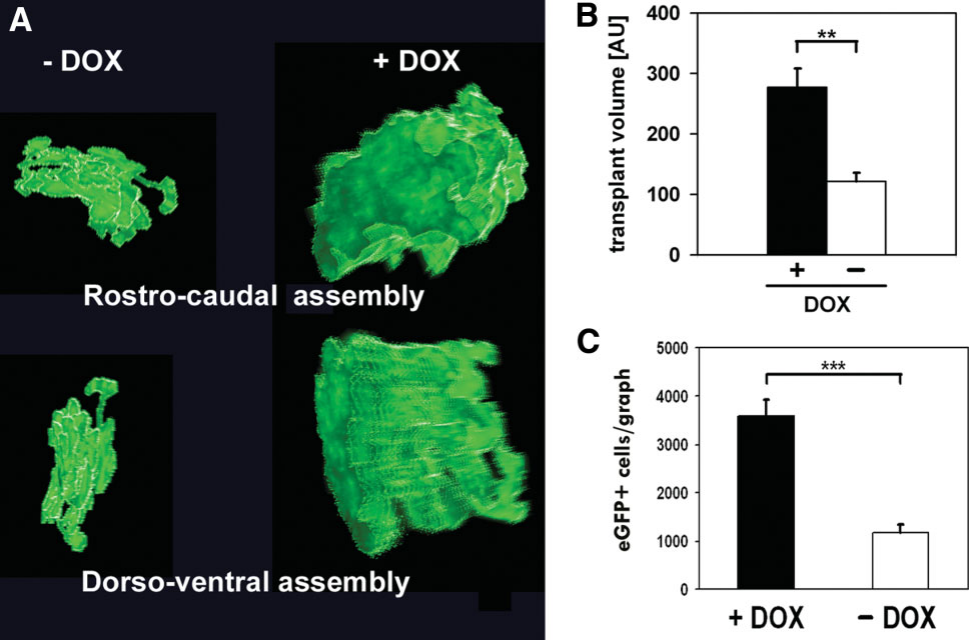
Increased graft size and cell survival after DOX-induced Runx1 expression. **(A):** Example of three-dimensional reconstructions of grafts of C57BL/6-β-actin (CAG)-eGFP:Sox10^+/rrTA^ boundary cap neural crest stem cells. **(B):** Quantification of graft size showing the relative increase in size after Runx1 overexpression (*p* = 5.5 × 10^−4^; *n* = 4). **(C):** Quantification of eGFP+ graft cell numbers showing an increased survival after 1 month in treated versus control (*p* = 1.4 × 10^−4^, *n* = 4). Abbreviations: DOX, doxycycline; eGFP, enhanced green fluorescent protein.

To evaluate transplant size and overall survival of transplanted bNCSCs as well as to rule out any contribution of endogenous cells to sensory neurogenesis, we generated bNCSCs from CAG-EGFP:Sox10^+/rtTA^ mouse embryos (ubiquitously expressing EGFP in all cells), transfected them with the TRE_bi_-Runx1-EYFP construct and transplanted them to the L4 DRG cavity as described earlier. The mean volume of DOX-treated transplants was 2.3 times larger than control (Fig. 6A, 6B; *p* = 5.5 × 10^−4^) and the total number of EGFP expressing cells in DOX-treated transplants was increased by 204% compared with control transplants (Fig. 6C; *p* = 1.4 × 10^−4^).

## DISCUSSION

In this study, we have shown that differentiation of bNCSCs towards subtype-specific sensory neuron phenotypes can be guided in *vitro* and *after* their engraftment into the DRG cavity with the Tet-On system.

For producing bNCSCs, we used DRGs and dorsal roots of 11.5-day old mouse embryos. At this stage, contaminating DRG progenitor cells are still present in the DRGs, but they cease to proliferate around E12 [35, 36]. The boundary cap cells proliferate throughout the entire embryogenesis [37], migrate to the DRGs and contribute to some trunk sensory neurons, satellite cells and Schwann cells [10, 12] It was shown previously that a rapidly amplifying population that could not be sub-cloned, (i.e., the non-stem cell DRG progenitor cells mentioned above) is lost at around 6–8 days of culture of boundary cap cells whereas after about 2 weeks of culture a more slowly dividing cell population appears that forms neurospheres and can be repeatedly subcloned without affecting its pluripotency [12]. Furthermore, it was shown that the DRG itself does not significantly contribute to neurosphere formation and that the bNCSCs (as well as the actual boundary cap cells in vivo) express Krox20, Sox10, and the multipotency marker SSEA-1, which is not the case for surrounding cell types [12, 13]. The BrdU labeling of neurons expressing the nociceptor-specific marker RET shows the presence of stem/progenitor boundary cap cells that continue to divide after transplantation. As the experiments in this study were performed with cells isolated in semiclonal conditions, however, it cannot be entirely excluded that a minority of cells in the bNCSC-population are indeed nonproliferative committed progenitors and that a small part of the effect might be attributed to selection in addition to the instructive role of Runx1.

In contrast to previously transplanted neurospheres derived from mouse [7] and human [8] forebrain, which did not express any specific neuronal markers after transplantation, in the experiments described here, we achieved differentiation of transplanted cells into neurons with extensive neurite outgrowth into the host dorsal roots and the expression of neuronal markers specific for the nociceptor neuron phenotype. Furthermore, DOX-treated recipients displayed increased graft size and cell survival. In vivo, a combination of intrinsic and extrinsic factors influences survival and differentiation of transplanted cells. Survival and differentiation of TrkA-expressing cells are dependent on nerve growth factor (NGF), whereas RET-expressing cells rely instead on members of the GDNF family [38, 39]. Differentiating immature sensory neurons may provide paracrine neurotrophic support in the transplant [40]. Non-neuronal cells, particularly from the host, are likely to be a major source of growth promoting factors, including NGF and GDNF [41, 42]. In collaboration with the Runx1-induced ectopic expression of the growth factor receptors TrkA and RET, these extrinsic factors may significantly contribute to survival and possibly also to differentiation of the DOX-treated, transplanted bNCSCs. Furthermore, recent studies have demonstrated that NGF promotes differentiation and maturation of nonpeptidergic neurons through both RET-dependent and RET-independent signaling mechanisms, possibly via Runx1 expression [39]. Specifically, we could observe upregulation of P2X3 in the transplants arguing that RET-independent markers are induced via the Runx1 pathway. This, in conjunction with the strong upregulation of RET would suggest that both types of nonpeptidergic genes are controlled by the Runx1 overexpression.

Compared with control transplants, we observed a significant increase in the number of TrkA+ cells as well as a reduction in the numbers of cells not expressing bTUB or GFAP in DOX-treated transplants, suggesting that Runx1 overexpression might be sufficient to drive initial differentiation. However, in the absence of a robust increase in the proportion of bTUB+ cells, our data suggest that this differentiation of grafted bNCSCs is to a large part glial. This is in accordance with previous in vitro data on Runx1 overexpression in bNCSCs promoting survival and neuronal maturation but not neurogenesis per se [32]. The same study showed that Runx1-induced cell survival of bNCSC-derived neurons is NGF-independent arguing for a more general effect of Runx1 on cell survival. However, since the grafted cells differentiate into more than one cell type it is not possible to discern between cell-autonomous effects of Runx1 and those involving signaling between the various differentiating cells in the transplant or with host cells. It is also possible that the increased amount of GFAP+ cells in the Runx1-overexpressing grafts may contribute to maintaining survival of neighboring neuronal precursors, reminiscent of the relationship between satellite cells and neurons in intact DRGs [43].

In the chick, it has been suggested that Runx1 directly activates TrkA transcription and that Runx1 overexpression in progenitors in vivo induces ectopic expression of TrkA [32]. In the same study, siRNA knock-down of early Runx1 expression as well as overexpression of a general dominant-negative Runx protein was shown to down-regulate TrkA expression and cause the subsequent death of the neurons. This suggests that Runx1 might have an effect on early progenitors by inducing TrkA expression in DRG precursors. There seems to be conflicting data with regards to an early role of Runx1 in the development of nociceptive sensory neurons since there is no loss of TrkA+ neurons in mice that lack Runx1 expression in DRG cells [26, 44]. Our findings indicate that ectopic Runx1 expression indirectly influences the survival of bNCSCs grafted into the DRG cavity.

The role of Runx1 in later stages of differentiation of DRG neurons has been convincingly demonstrated. Mice that lack Runx1 function selectively in the peripheral nervous system have an impaired perinatal switch from TrkA to RET of non-peptidergic nociceptors [45]. Indeed, in the mouse, from E14.5 to parturition, Runx1 expression is restricted to TrkA+ neurons, but by postnatal day 30, Runx1+ neurons are TrkA-negative and RET+ [26]. These two studies also show that Runx1 controls, via both repressing and activating gene expression, several aspects of maturation of nonpeptidergic neurons such as the concerted expression of specific ion channels and receptors. This includes the expression of the ATP-receptor P2X3. Indeed, after forced Runx1 expression, we observed a selective up-regulation of RET, IB4-binding, and P2X3 in differentiated bTUB+ neurons, as well as a decrease of RT97 and CGRP expression in the transplants.

The possibility that increased survival and differentiation to the specific type of neurons in the transplants was rather due to the DOX-treatment or to the electroporation procedure itself [46] was ruled out by the fact that none of the control transplants displayed any presence of nociceptor-specific markers. The latter appeared exclusively in Sox10-expressing cells that were transfected with an inducible Runx1 construct and treated with DOX. In agreement with this more mature and differentiated phenotype, DOX-treated Runx1-induced EGFP+ transplants displayed extensive neurite outgrowth to the dorsal roots towards the spinal cord.

Our data show that exogenously induced Runx1 expression does not affect the relative neuron/glia proportion in the transplants. There was a trend towards neuronal differentiation in DOX-treated transplants but the difference was not significant compared to DOX-untreated recipients. This finding suggests that the decision of neural progenitors to become neuronal or glial is regulated by other transcription factors in bNCSCs when they still express Sox10. The large proportion of RET+ cells is a bit surprising but might be due to a combination of RET being directly downstream of Runx1 [39] and that there is positive selection for bNCSCs that have been transfected and express Runx1.

The Sox10+/rtTA/TRE-Runx1 system used here appears to be sufficient to ensure advanced cell differentiation towards nociceptor subtype in vitro and in the transplants. The mechanism for this might be due to the specific targeting of Sox10-expressing cells for Runx1 overexpression. It was achieved by a new approach using the Tet-On system. The separation of the rtTA2^S^-M2 and TRE expressing vectors might serve as a useful tool to specifically target certain cell types for expression of the desired key transcription factor. It also gave us the possibility to mimic the normal cellular development pattern, in which the expression of the transcription factor Sox10 precedes that of Runx1, a temporal sequence which seems to be crucial for the guiding the differentiation of bNCSCs toward nociceptive-type sensory neurons.

## CONCLUSION

Our results demonstrate that using the Tet-system to exogenously control Runx1 expression in grafted bNCSCs resulted in their long-term survival, increased neurite outgrowth, and selective differentiation of the neuronal population toward a nonpeptidergic nociceptive neuronal phenotype. These results are achieved by cooperation between Runx1-induced intrinsic factors and extrinsic factors from the host environment. Our data suggest that the Tet-system can be successfully used in vivo to manipulate gene activation in transplanted cells. Hence, it is reasonable to speculate that timely activation to appropriate expression levels of just a few key transcription factors, specific for a certain type of stem/progenitor cell, would be sufficient to control survival and to guide the differentiation of transplanted cells to a desired cell type. Combining different Tet-system transactivators could allow the inducible expression of two genes [47] or, alternatively, the Tet-system could be used in combination with other conditional gene expression systems in mice [48, 49]. Concomitant or sequential expression of one or more key proteins can thus be implemented for directed differentiation of stem/progenitor cells, thus offering exciting new possibilities in understanding development and for treating diseases.

## DISCLOSURE OF POTENTIAL CONFLICTS OF INTEREST

The authors indicate no potential conflicts of interest.
